# PubChem3D: a new resource for scientists

**DOI:** 10.1186/1758-2946-3-32

**Published:** 2011-09-20

**Authors:** Evan E Bolton, Jie Chen, Sunghwan Kim, Lianyi Han, Siqian He, Wenyao Shi, Vahan Simonyan, Yan Sun, Paul A Thiessen, Jiyao Wang, Bo Yu, Jian Zhang, Stephen H Bryant

**Affiliations:** 1 National Center for Biotechnology Information, National Library of Medicine, National Institutes of Health, Department of Health and Human Services, 8600 Rockville Pike, Bethesda, MD 20894, USA

## Abstract

**Background:**

PubChem is an open repository for small molecules and their experimental biological activity. PubChem integrates and provides search, retrieval, visualization, analysis, and programmatic access tools in an effort to maximize the utility of contributed information. There are many diverse chemical structures with similar biological efficacies against targets available in PubChem that are difficult to interrelate using traditional 2-D similarity methods. A new layer called PubChem3D is added to PubChem to assist in this analysis.

**Description:**

PubChem generates a 3-D conformer model description for 92.3% of all records in the PubChem Compound database (when considering the parent compound of salts). Each of these conformer models is sampled to remove redundancy, guaranteeing a minimum (non-hydrogen atom pair-wise) RMSD between conformers. A diverse conformer ordering gives a maximal description of the conformational diversity of a molecule when only a subset of available conformers is used. A pre-computed search per compound record gives immediate access to a set of 3-D similar compounds (called "Similar Conformers") in PubChem and their respective superpositions. Systematic augmentation of PubChem resources to include a 3-D layer provides users with new capabilities to search, subset, visualize, analyze, and download data.

A series of retrospective studies help to demonstrate important connections between chemical structures and their biological function that are not obvious using 2-D similarity but are readily apparent by 3-D similarity.

**Conclusions:**

The addition of PubChem3D to the existing contents of PubChem is a considerable achievement, given the scope, scale, and the fact that the resource is publicly accessible and free. With the ability to uncover latent structure-activity relationships of chemical structures, while complementing 2-D similarity analysis approaches, PubChem3D represents a new resource for scientists to exploit when exploring the biological annotations in PubChem.

## Background

PubChem [1-4] (http://pubchem.ncbi.nlm.nih.gov) is an open repository for small molecules and their experimental biological activities. The primary goal of PubChem is to be a public resource containing comprehensive information on the biological activities of small molecules. PubChem provides search, retrieval, visualization, analysis, and programmatic access tools in an effort to maximize the utility of contributed information. The PubChem3D project adds a new layer to this infrastructure. In the most basic sense, PubChem3D [5-10] generates a 3-D conformer model description of the small molecules contained within the PubChem Compound database. This 3-D description can be employed to enhance existing PubChem search and analysis methodologies by means of 3-D similarity. Prior to PubChem3D, this similarity approach was limited to a 2-D dictionary-based fingerprint (ftp://ftp.ncbi.nlm.nih.gov/pubchem/specifications/pubchem_fingerprints.txt) to help relate chemical structures. With the advent of PubChem3D, this is now expanded to use a Gaussian-based similarity description of molecular shape [11-13] used in software packages such as ROCS [14] and OEShape [15] from OpenEye Scientific Software, Inc.

It is reasonable to ask, why do we consider 3-D similarity methodologies at all? To put it simply, 2-D methods, while very useful and far cheaper computationally, may not be enough. A pitfall of most 2-D similarity methods is a general lack of ability to relate chemically diverse molecules with similar biological efficacy and function. For example, if a small molecule adopts an appropriate 3-D shape and possesses compatible functional groups properly oriented in 3-D space, it will likely bind to the biological moiety of interest. This "lock and key" binding motif is a major premise of structure-based drug design, docking, and molecular modelling applied with varying degrees of success over the past twenty years or more [16-23]. These "compatible functional groups" involved in binding small molecules to proteins, which are typically used to define pharmacophores, are referred to here simply as "features". Therefore, in this context, 3-D similarity considering both shape and feature complementariness may be useful to find or relate chemical structures that may bind similarly to a protein target.

In its essence, 3-D similarity adds another dimension to data mining and it can provide some degree of orthogonality from 2-D similarity results. With 2-D similarity, one can typically see by eye increased changes in the chemical structure molecular graph with increasing dissimilarity [8,10]. With 3-D similarity, it is not always obvious by looking only at the molecular graph, often requiring one to visualize 3-D conformer alignments to relate diverse chemistries. In all, 3-D similarity is complementary to 2-D similarity and provides an easy-to-grasp understanding (*i.e.*, one can readily see by examining a conformer pair superposition that both shape and features are similar) that may help to provide a contrast or new insight to the same (biological) data.

This work gives an overview of the PubChem3D project and its current capabilities. The technology and background that allowed 3-D methodologies to be economically applied to the tens of millions of chemical structures in the PubChem Compound database are described elsewhere [5-10] covering various aspects of the project, including conformer model generation validation [6], the relative uniqueness of molecular shape [7], and 3-D neighboring methodology [8].

## Construction and Content

### 1. PubChem3D Coverage

As one can imagine, it does not make sense nor is it possible to compute a 3-D description for all chemical structures in PubChem (*e.g.*, complexes and mixtures). PubChem provides a 3-D conformer model description for each record in the PubChem Compound database that satisfies the following conditions:

(1) Not too large (with ≤ 50 non-hydrogen atoms).

(2) Not too flexible (with ≤ 15 rotatable bonds).

(3) Consists of only supported elements (H, C, N, O, F, Si, P, S, Cl, Br, and I).

(4) Has only a single covalent unit (*i.e.*, not a salt or a mixture).

(5) Contains only atom types recognized by the MMFF94s force field [24-26].

(6) Has fewer than six undefined atom or bond stereo centers.

**Figure 1:**
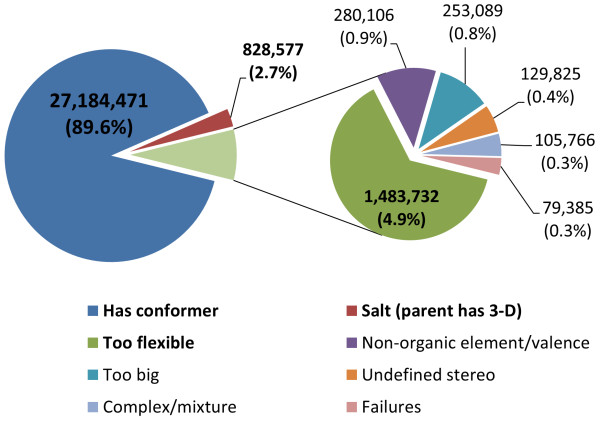
**PubChem Compound database 3-D coverage**. As one can see, 89.6% of all records have a 3-D conformer model. If one includes the parent compound of salts, this coverage can be considered to be 92.3%. Of the cases not having a 3-D conformer model, the majority are due to the flexibility of the chemical structure being too great to be suitable for conformer generation.

Figure 1 shows the PubChem3D coverage as of June 2011. Out of more than 30.3 million chemical structure records in the PubChem Compound database, there are nearly 27.2 million records with a 3-D description. This represents 89.6% of the PubChem Compound contents (92.3% when considering that 2.7% are salts whose parent structure has a 3-D description). Of the remaining 7.7% of chemical structures in PubChem devoid of a 3-D description, the largest category (representing 1.48 million or 4.9% of the total archive) consists of structures with more than 15 rotatable bonds. The next largest unique count (*i.e.*, those not already represented by structures with more than 15 rotatable bonds) is the cases of MMFF94s non-supported elements and non-supported atom environments (representing 280 thousand or 0.9% of the total archive, with an overlapping absolute count of 389 thousand). The remaining unique counts are the cases of large structures with +50 non-hydrogen atoms (representing 253 thousand or 0.8% of the total archive, with an overlapping absolute count of 882 thousand), excessive undefined stereo (representing 129 thousand or 0.4% of the total archive, with an overlapping absolute count of 234 thousand), chemical structures involving complexes or mixtures (representing 105 thousand or 0.3% of the total archive, with an overlapping absolute count of 324 thousand), and conformer generation failure (representing 79 thousand or 0.3% of the total archive). While the reasons for missing a 3-D description categories sometimes overlap, the ordering above is such that the one with the largest overall population is chosen first, with each subsequent category picking the largest remaining unique subpopulation not already covered, until all categories were exhausted.

### 2. Conformer Models

The computed coordinates for the 3-D representations are the essence of the PubChem3D project. Creation of the stored conformational models consists of multistep processes involving separate conformer generation, sampling, and post processing steps.

All conformers were generated by the OpenEye Scientific Software, Inc., OMEGA software [27-31] using the C++ interface, the MMFF94s force field [24-26] minus coulombic terms, and an energy filter of 25 kcal/mol. (Removal of coulombic terms [6,32-35] eliminated a bias towards conformations with energy-lowering intra-molecular interactions that tend not to be important for inter-molecular interactions, an important consideration given that the 3-D coordinates are generated in vacuo. Removal of attractive van der Waals terms did not have any noticeable effect [6].) A maximum of 100,000 conformers per chemical structure stereo isomer were allowed. When undefined stereo centers were present, each stereo isomer was enumerated and conformers independently generated. These stereo isomer conformers were then combined (2**5 = 32 maximum stereo permutations, 32 * 100,000 = maximum 3.2 million conformers).

Limiting to 100,000 conformations per stereo isomer can be a significant factor in limiting exploration of the conformational space. Ideally, one would want to explore the conformational space of a molecule exhaustively. In reality, it is not tractable to do so. For example, if one considers only three angles per rotatable bond and there are eleven rotatable bonds, this would yield 3**11 (= 177,147) possible conformers. If one considers four torsion angles per rotatable bond, and there are nine rotatable bonds, this would yield 4**9 (= 262,144) possible conformers. One can see how quickly systematic approaches can run into trouble with such exponential growth in the count of conformations and why there is a limit on how flexible a molecule is allowed to be.

With conformers generated, another important consideration is immediately obvious. It is not practical to store many thousands of conformers per compound. Therefore, after conformer generation is complete, the conformation count is reduced by sampling using root-mean-square-distance (RMSD) of pair-wise comparison of non-hydrogen atomic coordinates using the OEChem [36] OERMSD function with the automorph detection (which considers local symmetry equivalence of atoms such that, for example, rotation of a phenyl ring does not yield an artificially high RMSD) and overlay (which minimizes RMSD between conformers by rotation and translation of one conformer to the other) options selected. In some rare cases, the automorph detection was prohibitively expensive computationally and not used.

The sampling procedure employed is described elsewhere [7] but involves a two-stage clustering approach with an initial pass to partition-cluster conformers using an exclusion region hierarchy of decreasing dissimilarity (NlogN computational complexity, each cluster representative forms an exclusion region at a particular RMSD), followed by a step to remove edge-effects from the partition clustering (N^2 ^computational complexity using only the cluster representatives at the desired RMSD). The RMSD value used when sampling was dependent on the size and flexibility of the chemical structure.

**Equations 1 **and **2 **were developed [6] to help prevent using a conformer sampling RMSD that was less than the capability of the OMEGA software to reproduce bioactive ligand conformations. The equations were intended to ensure that 90% of the sampled conformer models of 25,972 small-molecule ligands, whose 3-D structures were experimentally determined, should contain at least one conformer within the RMSD sampling value to a bioactive conformation. The resulting *RMSD_pred *value was rounded to the nearest 0.2 increment. The smallest RMSD value used was 0.4. If more than 500 conformers resulted after sampling, the RMSD was incremented by a further 0.2 and the conformer model was re-clustered. This process was repeated as many times as necessary to restrict the overall count of conformers to be 500 or less.

(1) RMSD_pred= 0.219 + 0.0099 × nha+ 0.040 × er

where "*nha*" is the count of non-hydrogen atoms in the molecule, "*er*" is the effective rotor count, and "*RMSD_pred*" is the predicted average accuracy for a given "*nha*" and "*er*" value.

(2) er=rb+nara∕5

where "*er*" is the effective rotor count, "*rb*" is the rotatable bond count (computed using the OEChem "IsRotor" function) and "*nara*" is the count of non-aromatic ring atom count (OEChem OpenEye aromaticity model) excluding bridgehead atoms and SP2 hybridized atoms.

A post processing step was performed, after conformer model RMSD sampling, to completely relax the hydrogen atom locations by performing a full energy minimization where all non-hydrogen atoms were kept frozen. A subsequent "bump" check removed any conformers that had MMFF94 atom-atom interactions greater than 25 kcal/mol. Finally, each conformer was rotated and translated to their principal steric axes (*i.e.*, non-mass weighted principal moments of inertia axes) considering only non-hydrogen atoms.

It is important to note that the conformers produced are not stationary points on a potential energy hypersurface. In fact, one can readily achieve lower-energy conformations of a given chemical structure by performing an all-atom energy minimization to remove any bond, angle, or torsion strain present in vacuo. The PubChem3D conformer model for a chemical structure is meant to represent all possible biologically-relevant conformations that the molecule may have. In theory, one should have a reasonable chance to find any biologically accessible conformation within the RMSD sampling distance of the conformer model.

### 3. Conformer Model Properties

After a conformer model is produced, a series of properties are computed for each compound and each associated conformer. Table 1 lists the compound- and conformer-level properties provided by PubChem3D. The compound properties include: the sampling RMSD used to construct the conformer model; the MMFF94 partial charges per atom [36]; the functional group atoms that define each pharmacophore feature [15]; and the diverse conformer ordering, always starting with the default conformer per compound.

**Table 1:** PubChem3D properties and descriptors

	For each Compound	For each Conformer
MMFF94 partial charges	X	
Pharmacophore features	X	
Conformer model sampling RMSD	X	
Diverse conformer ordering	X	
Conformer identifier		X
MMFF94s Energy (minus coulombic terms)		X
Conformer volume		X
Steric shape moments		X
Shape self-overlap volume		X
Feature self-overlap volume		X
Shape fingerprint		X

Compound- and conformer-specific properties available for PubChem Compound records.

The feature definition lists the set of non-hydrogen atoms that comprise a given fictitious feature atom. The feature definitions are computed using the OEShape "ImplicitMillsDeans" forcefield [15,37]. Care is taken to (iteratively) merge feature definitions of common type that are within 1.0 Å distance of each other. Each feature definition is used to generate a fictitious "color" atom, whose 3-D coordinates are at the steric center of the atoms that comprise it (*i.e.*, at the average {X, Y, Z} value). There are six feature types used: anion, cation, (hydrogen-bond) acceptor, (hydrogen-bond) donor, hydrophobe, and ring.

The conformer properties include: the global conformer identifier (GID); conformer volume [15]; steric shape moments (monopole, quadrupole {Q_x_, Q_y_, Q_z_}, and octopole {O_xxx_, O_yyy_, O_zzz_, O_xxy_, O_xxz_, O_yyx_, O_yyz_, O_zzx_, O_zzy_, and O_xyz_}) [15]; shape self-overlap volume used in shape similarity computations [11]; feature self-overlap volume used in feature similarity computations [11]; MMFF94s energy with coulombic terms removed [38]; and the PubChem shape fingerprint [8].

(3) $$ST=\frac{V_{AB}}{V_{AA}+V_{BB}-V_{AB}}$$

where *ST *is the measure of shape similarity (shape Tanimoto), *V_AA _*and *V_BB _*are the respective self-overlap volume of conformers A and B, and *V_AB _*is the common overlap volume between them.

(4) $$CT=\frac{\underset{f}{\sum }V_{AB}^{f}}{\underset{f}{\sum }V_{AA}^{f}+\underset{f}{\sum }V_{BB}^{f}-\underset{f}{\sum }V_{AB}^{f}}$$

where *CT *is the measure of feature similarity (color Tanimoto), the index "*f*" indicates any of the six independent fictitious feature atom types, $V_{AA}^{f}$ and $V_{BB}^{f}$ are the respective self-overlap volumes of conformers A and B for feature atom type *f*, and $V_{AB}^{f}$ is the overlap volume of conformers A and B for feature type *f*.

(5) ComboT=ST+CT

where *ComboT *is the combo Tanimoto, *ST *is the shape Tanimoto, and *CT *is the color Tanimoto.

A diverse ordering of conformers is provided for each compound conformer ensemble [8,39,40]. Using the lowest energy conformer in the ensemble as the initial default conformer, the conformer most dissimilar to the first is selected as the second diverse conformer. The conformer most dissimilar to the first two dissimilar conformers is chosen as the third diverse conformer. This process is repeated until there are no more conformers to be assigned a dissimilarity ordering. Similarity is measured by ST (**Equation 3**) and CT (**Equation 4**), involving a conformer superposition optimization [11,36] to maximize the shape volume overlap between two conformers by means of rotation and translation of one conformer to the other. This is followed by a single point CT computation at the ST-optimized conformer pair overlay. The ST and CT are then added to yield a combo Tanimoto (**Equation 5**). The conformer with the smallest sum of combo Tanimoto to all *assigned *dissimilar conformers is selected as the next most dissimilar. In the case of a tie, the one with the largest sum of combo Tanimoto to *unassigned *conformers is used.

Note that PubChem has another source of 3-D information of small molecules, besides PubChem3D. The PubChem Substance database (unique identifier: SID) contains 3-D structures of small molecules deposited from individual depositors, which can be either experimentally determined or computationally predicted. For clarification, these depositor-provided structures are called "*substance *conformers", and the theoretical conformers generated by PubChem3D for each PubChem Compound record (unique identifier: CID) are called "*compound *conformers". For an efficient use of the PubChem3D resources, it is necessary to assign a unique identifier to each of compound conformers in the PubChem Compound database and substance conformers in the PubChem Substance database. The global conformer identifier (GID) uniquely identifies each conformer and is stored as a hex-encoded 64-bit unsigned integer, where the first 16-bits (0x000000000000FFFF) correspond to the local conformer identifier (LID), which is specific to a given conformer ensemble, the next 16-bits (0x00000000FFFF0000) are the version identifier (always zero for PubChem3D compound conformers, but nonzero for deposited substance conformers) and the last 32 bits (0xFFFFFFFF00000000) correspond to the structure identifier. This identifier is a compound identifier (CID), if the version identifier is zero, and a substance identifier (SID), when the version identifier is non-zero (the version identifier indicates the substance version to which the conformer corresponds). Substance conformer identifiers allow deposited 3-D coordinates to be utilized effectively by the PubChem3D system. As one can see, the GID provides global conformer identification system across all PubChem conformers.

A shape fingerprint is computed for the first ten diverse conformers. To generate this property, each conformer is ST-optimized to a set of reference conformers that describe the entire shape space diversity of the contents of PubChem3D. If the conformer is shape similar beyond a particular threshold to a reference conformer, the reference conformer identifier (CID and LID) and a packed rotational/translational matrix (64-bit integer) are retained. This makes each set reference conformer like a bit in a binary fingerprint, however; in this case, additional information (the superposition) is also retained. One can imagine that these shape fingerprints are a little like coordinates in shape space, mapping where a given conformer is located.

This shape fingerprint can be used in several ways during 3-D similarity computation and was born out of our earlier research [8,41] on "alignment recycling." This work demonstrated that similar conformers align to a reference shape in a similar way. This means that, if one is interested only in finding similar shapes, conformer pairs that do not have common shape fingerprint "bits" can be ignored (*i.e.*, there is no need to perform a computationally intensive conformer alignment overlap optimization between two conformers when no common shape fingerprint reference exists, because the two conformer shapes are dissimilar to the extent that they may not need to be considered further). Additionally, when a common shape fingerprint reference exists between two conformers, one can "replay" the alignments of the two conformers to the common reference shape to yield a conformer alignment overlap between conformers that is (typically) very close to the optimal overlay; thus speeding up any conformer alignment overlap optimization but also providing an opportunity to further skip overlap optimization, when the best pre-optimized alignment overlap is not sufficient.

### 4. Similar Conformer Neighboring Relationship

Analogous to the precomputed "Similar Compounds" relationship for 2-D similarity, PubChem3D now provides a "Similar Conformers" neighboring relationship [8] using 3-D similarity. This neighboring takes into account both conformer shape similarity and conformer pharmacophore feature similarity. Essentially, this is equivalent to performing a shape-optimized similarity search using ROCS [14,15] at a threshold of ST > 0.795 and CT > 0.495, when both conformers have defined pharmacophore features. To allow for compounds devoid of features to be neighbored, a threshold of ST > 0.925 is used, but with the caveat that both conformers must not have any defined pharmacophore features. Currently, three diverse conformers per compound are neighbored; however, this may change, with up to ten conformers per compound used as computational resources allow. The conformers used for neighboring correspond to the first "N" conformers in the diverse conformer list property. (See the **Conformer Model Properties **section.) This ensures maximal coverage of the unique shape/feature space of a chemical structure as additional conformers are considered in neighboring.

### 5. FTP Site

PubChem3D data is available on the PubChem FTP site (ftp://ftp.ncbi.nlm.nih.gov/pubchem/Compound_3D). One may download in bulk 3-D descriptions of PubChem Compound records. On average there are approximately 110 conformers per compound in the PubChem3D system; however, not all data is provided for public download, in part due to the overall size being many terabytes, more data than one can readily share publicly. Therefore, two different subsets are provided in various file formats (SDF, XML, and ASN.1) that correspond to either the default conformer or the first ten conformers in the diverse conformer list property. (See the **Conformer Model Properties **section.) Beyond these two conformer subsets of PubChem3D, one may also find a description of the conformers that comprise the PubChem3D shape fingerprint. These conformers represent all shape diversity present in the PubChem3D system for a given analytic volume range and a given level of shape similarity ST threshold.

The "Similar Conformers" neighboring relationship is also provided for download. This conformer pair relationship (one per line) includes the respective conformer identifiers, ST, CT, and the 3 × 3 rotation matrix and translation vector (applied in that order) to superimpose the second conformer to the first. The rotation/translation refers to the coordinates provided in the download set of ten diverse conformers or otherwise available for download from our PubChem download facility. (See the **Utility: Download **section.)

## Utility

### 1. NCBI Entrez Interface

The primary search interface for PubChem is Entrez [4], *e.g.*, for the PubChem Compound database, accessible by means of the PubChem homepage (http://pubchem.ncbi.nlm.nih.gov) or the URL: http://www.ncbi.nlm.nih.gov/pccompound?Db=pccompound. There are fourteen Entrez indexes available to query PubChem Compound records based on 3-D information detailed in Table 2. For example, to find which compound conformer models were sampled in the RMSD range between 0.4 and 0.6, one would perform the query "0.4:0.6[ConformerModelRmsd3D]".

**Table 2:** PubChem3D Entrez indexes

Index name	Description and example query
ConformerCount3D	Conformer count (*e.g.*, for compounds with only 1-5 conformers in their ensemble: "1:5[ConformerCount3D]")
ConformerModelRmsd3D	Conformer sampling RMSD in Å (*e.g.*, for compounds with 0.4 Å sampling RMSD: "0.4[ConformerModelRmsd3D]")
EffectiveRotorCount3D	Effective rotor count (*e.g.*, for compounds with 5.2-5.6 effective rotors: "5.2:5.6[EffectiveRotorCount3D]")
FeatureCount3D	Total count of individual features (*e.g.*, for compounds with 0-4 total features: "0:4[FeatureCount3D]")
FeatureAcceptorCount3D	Hydrogen-bond acceptor count (*e.g.*, for compounds with three acceptor features: "3[FeatureAcceptorCount3D]")
FeatureAnionCount3D	Anion count at pH 7 (*e.g.*, for compounds with 0-1 anion features: "0:1[FeatureAnionCount3D]")
FeatureCationCount3D	Cation count at pH 7 (*e.g.*, for compounds with one cation feature: "1[FeatureCationCount3D]")
FeatureDonorCount3D	Hydrogen-bond donor count (*e.g.*, for compounds with 5-11 donor features: "5:11[FeatureDonorCount3D]")
FeatureHydrophobeCount3D	Hydrophobe count (*e.g.*, for compounds with no hydrophobe features: "0[FeatureHydrophobeCount3D]")
FeatureRingCount3D	Ring count (*e.g.*, for compounds with 1-2 ring features: "1:2[FeatureRingCount3D]")
Volume3D	Analytic volume of the first diverse conformer (default conformer) for each compound (*e.g.*, for volume range 110-220.5 Å^3^: "110:220.5[Volume3D]")
XStericQuadrupole3D	Q_x _(describes length) of the first diverse conformer (default conformer) for each compound (*e.g.*, for Q_x _range 20.2-30.4 Å^5^: "20.2:30.4[XStericQuadrupole3D]")
YStericQuadrupole3D	Q_y _(describes width) of the first diverse conformer (default conformer) for each compound (*e.g.*, for Q_y _range 5.1-9.8 Å^5^: "5.1:9.8[YStericQuadrupole3D]")
ZStericQuadrupole3D	Q_z _(describes height) of the first diverse conformer (default conformer) for each compound (*e.g.*, for Q_z _range 1.3-4.2 Å^5^: "1.3:4.2[ZStericQuadrupole3D]")

Searchable compound- and conformer-specific fields available for PubChem Compound records.

The indexes for "Volume3D", "XStericQuadrupole3D", "YStericQuadrupole3D", and "ZStericQuadrupole3D" correspond, respectively, to the analytic volume and the three steric quadrupole moments [9,12,42] for only the first conformer in the diverse conformer list (*i.e.*, the default conformer). The steric quadrupoles essentially correspond to the extents of the compound, where X, Y, and Z correspond to the length, width, and height. For example, to find very long, near-linear compounds, one may give the PubChem Compound Entrez query "50:100[XStericQuadrupole3D] AND 0:1[YStericQuadrupole3D] AND 0:1[ZStericQuadrupole3D]". Please note that shortcuts exist for most indexes. These are documented in the PubChem Help "PubChem Indexes and Filters in Entrez" section (http://pubchem.ncbi.nlm.nih.gov/help.html#PubChem_index).

PubChem also provides filtering capabilities. Unlike indexes, which hold discrete values, filters are Boolean-based (*i.e.*, either a record is in the list or it is not). PubChem3D provides some additional filtering capabilities. In the case of the PubChem Compound database, there is a filter "has 3d conformer" that will indicate whether a given compound record has a 3-D conformer model by means of the PubChem Compound query: ""has 3d conformer"[filter]".

Filtering capabilities were also expanded in the PubChem Substance database. Two filters were added: "has deposited 3d" and "has deposited 3d experimental" to indicate when a substance record has 3-D coordinates and when the contributed 3-D coordinates were determined experimentally, respectively. For example, to find all experimentally determined 3-D structures for substance records, one would use the PubChem Substance databases query: ""has deposited 3d experimental"[filter]".

### 2. Visualization

**Figure 2:**
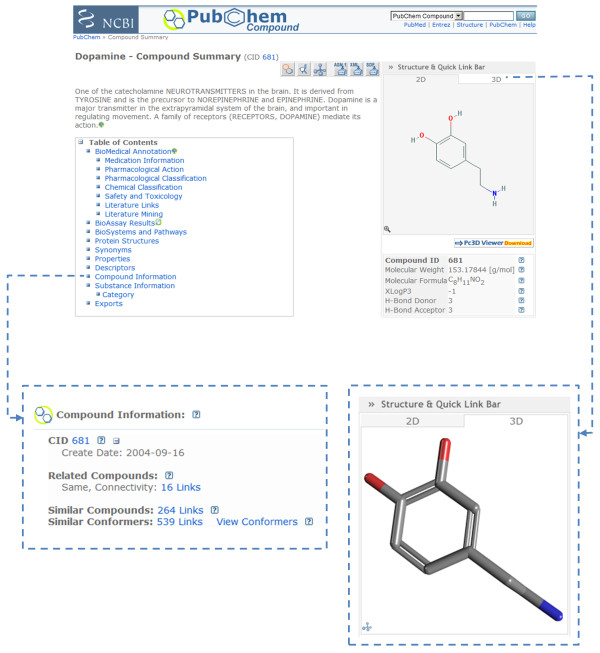
**Summary page enhancements**. A snapshot of the PubChem Compound summary page of dopamine (CID 681). Clicking on the "3D" tab on the right side of the page shows the 3-D structure of the molecule. Clicking the "Compound information" in the "Table of Contents" box directs users to 2-D neighbors ("Similar Compounds") and 3-D neighbors ("Similar Conformers").

**Figure 3:**
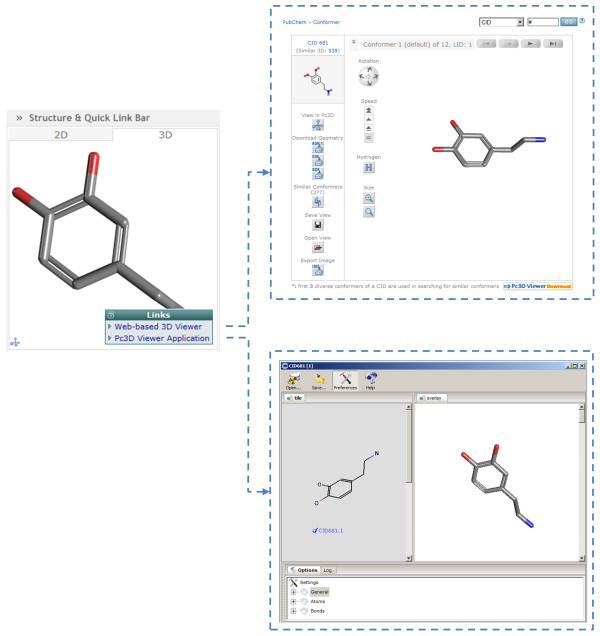
**Visualization of a 3-D structure conformer**. Clicking on the 3-D image on the PubChem Compound summary page (left) shows links to the web-based 3-D viewer (top right) and the Pc3D desktop helper application (bottom right).

Each PubChem Compound (and Substance) record has a summary page as depicted in Figure 2 (http://pubchem.ncbi.nlm.nih.gov/summary/summary.cgi?cid=681 for dopamine). When a 3-D conformer model can be produced for a compound record (or a depositor-provided 3-D coordinates for the substance record), a 3-D image of the structure will be available by clicking the "3D" tab. In the case of a PubChem Compound record, this corresponds to the first diverse conformer, which is the default conformer. As shown in Figure 3, if one clicks the image, a popup menu appears allowing one to invoke the "Web-based 3D Viewer" or to send the 3-D information to the "Pc3D Viewer Application".

The Pc3D viewer application can be downloaded and installed on PC, Mac, or Linux computers. A link to download this application can be found below the image on a given summary page or other PubChem3D aware pages (*e.g.*, see the "Pc3D Viewer Download" icon in Figure 2). The viewer provides an interface for rendering 3-D structures of PubChem Compound records and visualizing their superpositions. With a customizable 3-D rendering engine that provides dynamic molecular visualization experience, it has the ability to create high-resolution, publication-quality images. It allows use of XYZ model files and SDF files and supports PubChem native formatted files (with the .pc3d or .asn extension).

**Figure 4:**
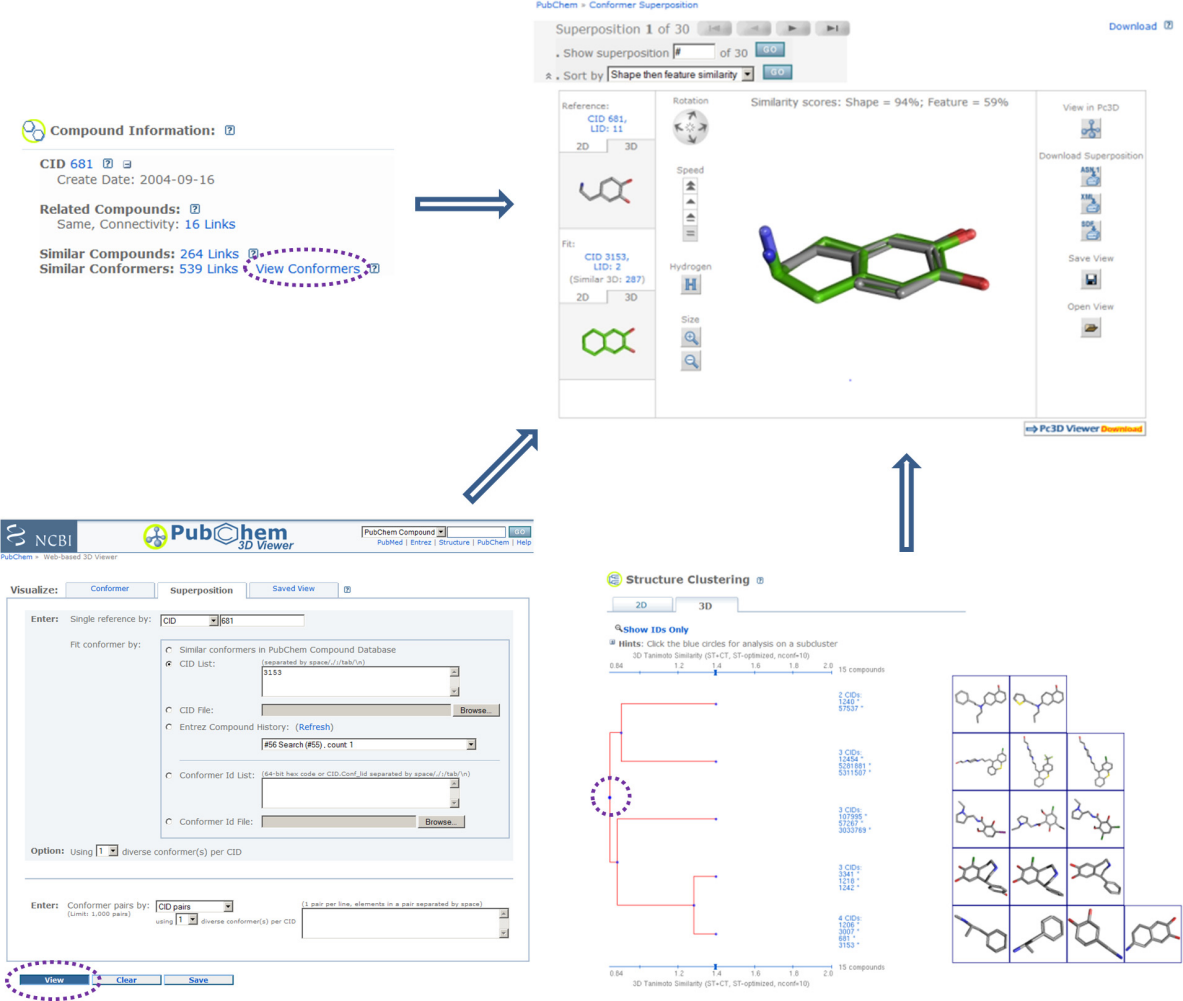
**Visualization of 3-D structure conformer superpositions**. Superpositions between compound conformers are accessible from various PubChem3D-aware applications. The PubChem Compound summary page (top left) allows the "Similar Conformers" neighboring relationship to be visualized. The PubChem3D web-based viewer (bottom left) allows arbitrary superpositions to be generated. The PubChem Structure Clustering tool (bottom right) enables all pair-wise superpositions to be examined.

The web-based 3-D viewer, like the Pc3D viewer application, allows one to browse 3-D conformers available for substances or compounds and their superpositions. This interactive tool (accessible via http://pubchem.ncbi.nlm.nih.gov/vw3d/) operates without the need for a web browser plug-in (and does not use Java, for support related reasons) by means of displaying a series of images to simulate molecule rotation. As shown in Figure 4, besides providing immediate access to the "Similar Conformer" neighboring relationship per compound (and per compound conformer), users can access various controls to perform such tasks as: superposition or conformer navigation, data export, conformer rotation type, conformer rotation speed, conformer image resize, conformer filtering, and sorting. The viewer allows any arbitrary set of 3-D compound conformers or conformer pairs (substance and compound) that exists within PubChem to be viewed or superimposed. This tool is also the primary resource to visualize and manage 3-D information from various PubChem3D-aware tools, including 3-D conformer search and 3-D structure clustering.

### 3. Search

The PubChem Structure Search system [1] (accessible via http://pubchem.ncbi.nlm.nih.gov/search/) allows one to search the PubChem Compound database using a chemical structure in various formats. PubChem3D adds a new capability to this system by allowing one to perform a 3-D similarity search and to visualize the results. At the time of writing, this similarity search is essentially equivalent to that described in the **Similar Conformer Neighboring Relationship **section. If 3-D coordinates are not provided for a chemical structure query, they are generated automatically, as-is possible, while keeping in mind that not all chemical structures can be covered by the PubChem3D system. (See the **PubChem3D Coverage **section for more details.) To aid in performing automated queries, a programmatic interface is available. (See the **Programmatic Interface **section for more details.)

A 3-D conformer search currently considers the first three diverse conformers per compound as candidates for "Similar Conformers". (See diverse conformer ordering in the **Conformer Model Properties **section.) Given that there are more than 27 million CIDs and three conformers per compound are being considered, this means that there are around 81 million conformers considered by each 3-D query. This count will change as a function of time as data is added to PubChem and as the count of conformers per compound are increased. To achieve adequate query throughput, an "embarrassingly parallel divide-and-conquer" strategy is employed. The PubChem Compound conformer data set is subdivided into multiple evenly-sized subsets. Each subset is then searched in parallel. If more query throughput is desired, and the computational capacity exists, the solution is simple; one simply needs to increase the count of evenly-sized subsets to simultaneously process.

### 4. Download

The PubChem Download facility [1] (http://pubchem.ncbi.nlm.nih.gov/pc_fetch) allows one to download PubChem records resulting from a search or a user-provided identifier list. With the advent of the PubChem3D layer, there is now the ability to download up to ten diverse conformers per compound. Alternatively, 3-D images may be downloaded (for the default conformer, only). A programmatic interface is available. (See the **Programmatic Interface **section for more details.)

### 5. Similarity Computation

The PubChem Score Matrix facility (http://pubchem.ncbi.nlm.nih.gov/score_matrix) allows one to compute pairwise similarities of a set of PubChem compound records (up to 1,000,000 similarity pairs per request). The PubChem3D layer adds the ability to compute 3-D similarities using up to ten conformers (either the first *N*-diverse conformers or a user-provided conformer set) per compound per request. Additionally, this service allows one to select the type of superposition optimization (shape or feature) to perform. A programmatic interface is available. (See the **Programmatic Interface **section.)

### 6. Clustering and Analysis

The PubChem Structure Clustering tool [10] (http://pubchem.ncbi.nlm.nih.gov/assay/assay.cgi?p=clustering) allows one to perform single-linkage clustering for up to 4,000 compounds at a time. This interactive tool provides visualization, subset, selection, and analysis capabilities. For example, the dendrogram allows compounds to be grouped into clusters by clicking the Tanimoto bar provided above and below the dendrogram (see the bottom right panel in Figure 4). One can then click on the cluster to view the individual compounds or perform other operations. The PubChem3D layer adds the ability to cluster compounds according to their 3-D similarities, with up to ten diverse conformers per compound. This service allows one to select: the superposition optimization type (shape or feature); whether to cluster all conformers or just the most similar conformer pair; and the conformer similarity metric.

### 7. Programmatic Interface

PubChem provides a programmatic interface called the Power User Gateway (PUG) [1]. This extends the capabilities provided by the NCBI eUtils programmatic interface [43], which interfaces the NCBI Entrez search engine contents. PUG can be used to send programmatic requests (*e.g.*, to perform queries or other tasks). If a request does not complete, a request ID is returned. One uses this to "poll" whether the request is completed, at which point an URL is provided to obtain the results. This is necessary, considering that most user requests are queued and may not be executed or completed immediately. A PUG/SOAP interface exists to allow the SOAP-based protocol to be used to route requests. SOAP-interfaces are readily available for most programming (*e.g.*, Java, C#, VisualBasic) and scripting languages (*e.g.*, Perl, Python), as well as workflow applications (*e.g.*, Taverna [44], Pipeline Pilot [45]). The PubChem3D layer extensions are now available in individual PUG-aware interfaces and by means of the PUG/SOAP interface.

## Examples of use

To assist in understanding how PubChem3D can be useful to locate additional biological annotation and enhance one's ability to identify potential structure-activity relationships, a series of illustrative examples were prepared. These examples benefit from a recent study [10] of the statistical distribution of random 3-D similarities of more than 740,000 biologically tested small molecules in PubChem using a single conformer per compound, where the average (μ) and standard deviation (σ) of the shape-optimized ST, CT, and ComboT scores between two randomly selected conformers were found to be 0.54 ± 0.10, 0.07 ± 0.05, and 0.62 ± 0.13, respectively. The probability of two random conformers having a ST-optimized similarity score greater than or equal to the μ + 2σ threshold (*i.e.*, 0.74, 0.17, and 0.88 for ST, CT, and ComboT, respectively) was 2%, 4%, and 3% for ST, CT, and ComboT, respectively. This statistical information is meaningful to provide reasonable 3-D similarity thresholds, whereby one can be confident that most of the 3-D similarities between chemical structures is not simply by chance. When a group of chemical structures with similar biological activity and function are shown to have 3-D similarity to each other above these thresholds, it suggests that a common macromolecule binding interaction orientation exists and, furthermore, that the features required for such binding are present.

### 1. Finding additional biological annotation

**Figure 5:**
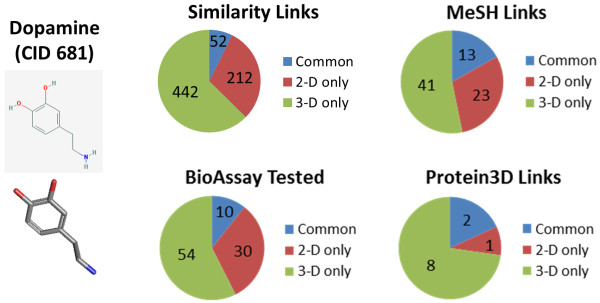
**3-D similarity relationship finds additional biological annotation**. Comparison of the 2-D "Similar Compound" and 3-D "Similar Conformer" neighboring relationships using dopamine to demonstrate how both neighboring relationship complement each other when locating related chemical structures with unique biological annotation.

In a data system such as PubChem, with a very uneven amount of biological annotation, it is helpful to find related chemical structures where more information is known. PubChem provides two precomputed neighboring relationships to locate similar chemical structures. The "Similar Conformers" neighboring relationship precomputes the 3-D similarity between all chemical structures in PubChem, while the "Similar Compounds" neighboring relationship precomputes the 2-D similarity. Using dopamine (CID 681) as an example, Figure 5 shows there can be relatively little commonality between 2-D and 3-D similarities; however, both relationships find chemicals that are related, with the 2-D similarity being good at finding chemical analogs of a given chemical while the 3-D similarity is skilled at locating molecules with similar shape and similar 3-D orientation of binding features. Therefore, use of both neighboring relationships allows a larger number of related chemicals to be found with associated biomedical literature (MeSH Links), biologically tested (BioAssay Tested), or bound to a protein 3-D structure (Protein3D Links).

### 2. Relating chemical probes for same biological target

**Figure 6:**
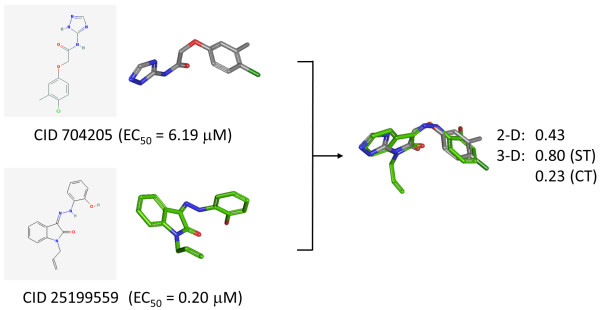
**Relating biologically active compounds by means of PubChem3D**. Chemical probes ML088 (CID 704205) and ML087 (CID 25199559) from PubChem BioAssay 1548 against tissue non-specific alkaline phosphatase (TNAP, GI:116734717) are not similar by 2-D similarity but are by 3-D similarity.

ML088 (CID 704205) and ML087 (CID 25199559), shown in Figure 6, are chemical probes reported [46] in a PubChem BioAssay (AID 1548) with EC50s of 6.19 μM and 0.20 μM, respectively. Both probes target a common protein, the tissue non-specific alkaline phosphatase (TNAP, GI 116734717), the deficiency of which is associated with defective bone mineralization in the form of rickets and osteomalacia. At first glance, these two chemical structures are rather dissimilar, with a 2-D subgraph similarity of 0.43 using the PubChem fingerprint. This suggests the two chemical structures are unrelated to each other, giving no hint as to why they have similar biological function and efficacy. Using 3-D similarity, by means of the PubChem3D web-based viewer as shown in Figure 6, the shape, feature, and combo similarities (0.80, 0.23, and 1.03 for ST, CT, and ComboT, respectively) tell a very different story. The two chemical structures are 3-D similar, suggesting that the two chemical structures can adopt a similar shape and have some binding features in a common 3-D orientation, thus helping to relate the observed biological activity by providing a hypothesis that the two inhibitors may bind in a similar manner. While this could be interpreted as simply highlighting a deficiency in the PubChem 2-D similarity metric, in this case, PubChem 3-D similarity complements the PubChem 2-D similarity by allowing such a similarity relationship to be found between these two chemical probes.

### 3. Relating chemically diverse structures with same pharmacological action

**Figure 7:**
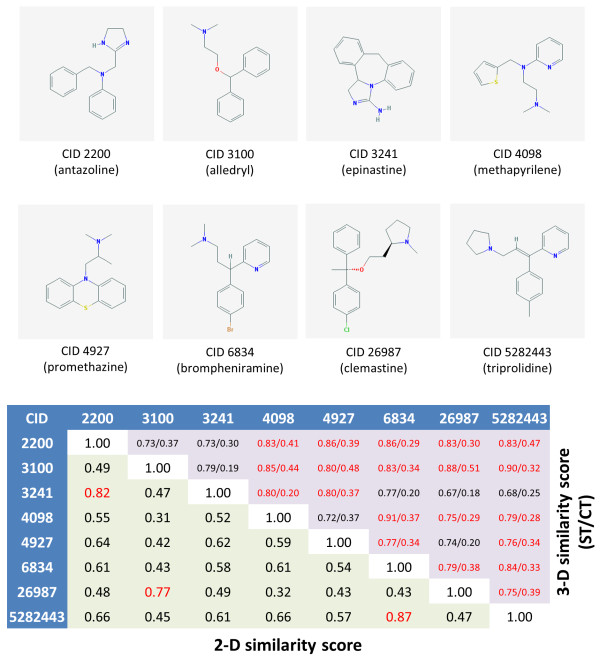
**Similarity score matrix for selected histamine H1 receptor antagonist anti-inflammatory drugs**. The lower triangle of the score matrix corresponds to the 2-D similarity computed using the PubChem fingerprint. The upper triangle corresponds to the 3-D similarity ST/CT scores. The matrix elements in red text indicate a 2-D similarity ≥ 0.75 or 3-D similarity with ST ≥ 0.74 and ComboT ≥ 1.0. The first ten diverse conformers per molecule were superimposed using shape-based optimization and the single conformer-pair per compound-pair with the largest ComboT retained.

**Figure 8:**
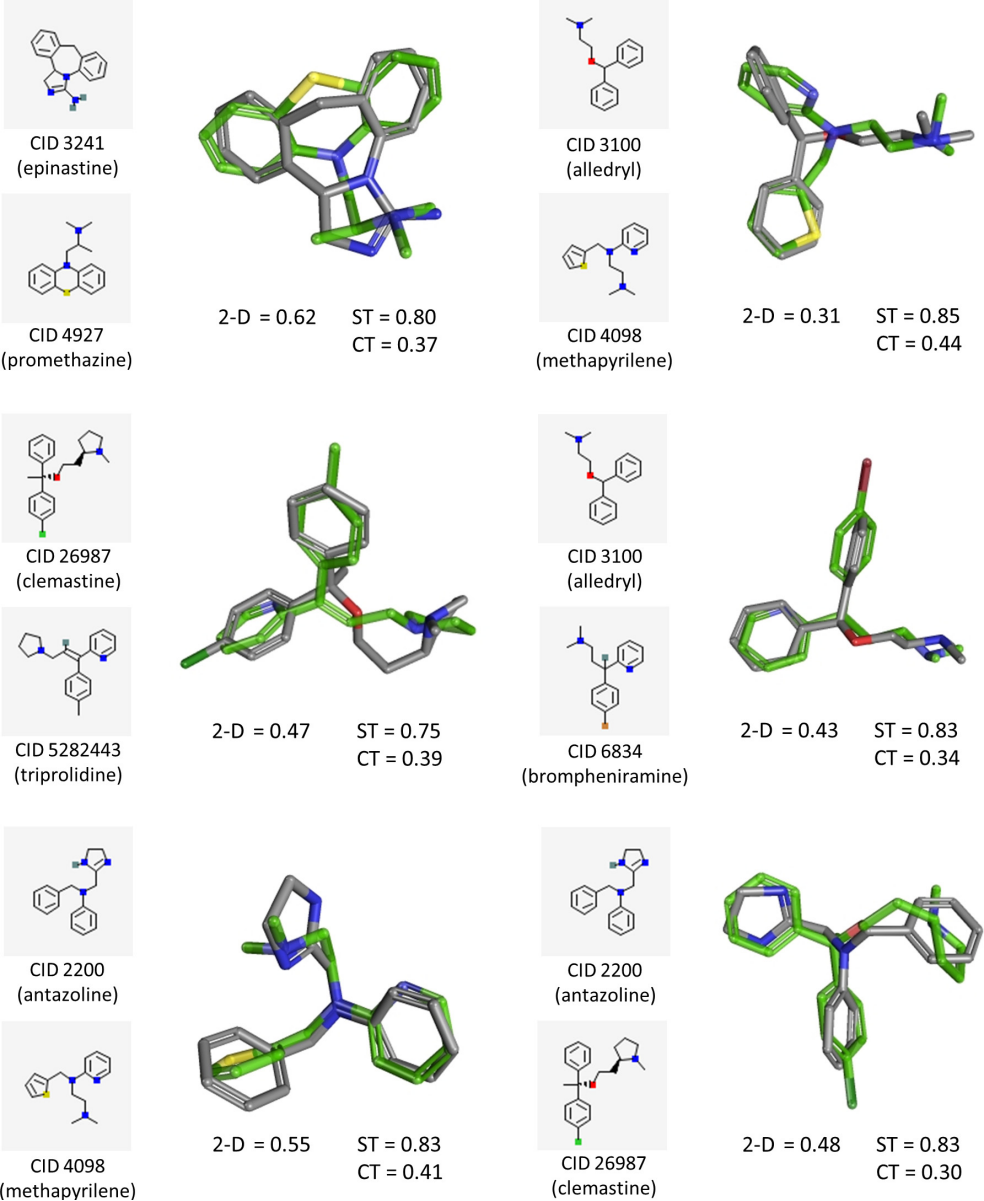
**3-D superposition of selected histamine H1 receptor antagonist anti-inflammatory drugs**. Although there is little 2-D similarity, using the PubChem fingerprint, substantial 3-D similarity is found between various structurally diverse anti-inflammatory drugs.

Figure 7 shows the 2-D and 3-D similarity score matrices for a carefully selected set of eight anti-inflammatory drug molecules having the same MeSH [47] pharmacological action annotation of "Histamine H1 Antagonists" (MeSH ID 68006634). Figure 8 depicts a subset of 3-D ST-optimized superpositions resulting from the 28 unique compound pairs. The 2-D Tanimoto similarity values between these compounds are quite low, with only three compound pairs above ≥ 0.75, indicating that the 2-D similarity method based on the PubChem fingerprint fails to interrelate their common biological activity as histamine H1 receptor antagonists. On the contrary, the 3-D similarity between these eight molecules is rather high, with a ST ≥ 0.74 and ComboT ≥ 1.0 for all but eight of the 28 compound pairs. As illustrated in Figure 8, even if the 2-D Tanimoto value between a pair of molecules is as low as 0.31, they can still have significant structural overlap in 3-D shape/feature space, resulting in relatively larger ST and CT similarity scores. The structure clustering tool is specifically geared towards helping to identify such structure-activity trends in 3-D similarity (as well as 2-D similarity) space and, in combination with the PubChem3D viewer, allow them to be visualized. If one thinks about this, it shows how easy it might be to "scaffold hop" or relate diverse chemical structures with similar biological function by examining 3-D similar chemicals in PubChem. It may also suggest that one may be able to better understand additional biological functions of known drugs (*i.e.*, so called "side effects") by examining their PubChem 3-D similarity to other chemicals with known biological roles.

## Conclusions

A new resource for scientists, PubChem3D, layered on top of PubChem, provides a new dimension to its ability to search, subset, export, visualize, and analyze chemical structures and their associated biological data. With a broad suite of tools and capabilities, 3-D similarity is given equal footing to assist in finding non-obvious trends in experimentally observed biological activity. As a complement to 2-D similarity, 3-D similarity demonstrates a capability to relate chemical series that are not sufficiently 2-D similar.

## Abbreviations

2-D: (2-dimensional); 3-D: (3-dimensional); MMFF: (Merck Molecular Force Field); RMSD: (root-mean-square distance).

## Competing interests

The authors declare that they have no competing interests.

## Authors' contributions

All authors contributed in a material way to the PubChem3D project. Specific attributable contributions are as follows: EEB drafted the manuscript and performed all major project aspects not directly attributed to others; JC implemented the web-based viewer and search interfaces; SK drafted the manuscript and helped develop neighboring accelerators; LH and YS developed analysis database components; WS and SH developed storage and neighboring database components; VS developed the image generation methodology, PC3D viewer application, search backends, and viewer backends; PAT developed the download and score matrix facilities; JW developed the structure clustering and heat-map facilities; BY and PAT developed the identifier exchange facilities; JZ developed the compound/substance summary facilities; and SHB heads the PubChem project. All authors read and approved the final manuscript.
